# Time on Therapy for at Least Three Months Correlates with Overall Survival in Metastatic Renal Cell Carcinoma

**DOI:** 10.3390/cancers11071000

**Published:** 2019-07-17

**Authors:** Viola J. Chen, Gabriela Hernandez-Meza, Prashasti Agrawal, Chiyuan A. Zhang, Lijia Xie, Cynthia L. Gong, Christian R. Hoerner, Sandy Srinivas, Eric K. Oermann, Alice C. Fan

**Affiliations:** 1 Division of Oncology, Department of Medicine, Stanford University School of Medicine, Stanford, CA 94305, USA; 2 Icahn School of Medicine at Mount Sinai, New York, NY 10029, USA; 3 Department of Medicine, Stanford University School of Medicine, Stanford, CA 94305, USA; 4 Department of Urology, Stanford University School of Medicine, Stanford, CA 94305, USA; 5 Department of Medicine, Highland Hospital, Oakland, CA 94602, USA; 6 Division of Neonatology and the Fetal and Neonatal Institute, Children’s Hospital Los Angeles (CHLA), Department of Pediatrics, Keck School of Medicine, University of Southern California, Los Angeles, CA 90033, USA; 7 Department of Neurosurgery, Icahn School of Medicine at Mount Sinai, New York, NY 10029, USA

**Keywords:** metastatic renal cell carcinoma, targeted kinase inhibitors, immunotherapy, systemic treatment, therapy sequencing, kidney cancer, RCC, IMDC criteria, favorable-, poor-, and intermediate-risk prognosis RCC

## Abstract

With 15 drugs currently approved for the treatment of metastatic renal cell carcinoma (mRCC) and even more combination regimens with immunotherapy on the horizon, there remains a distinct lack of molecular biomarkers for therapeutic efficacy. Our study reports on real-world clinical outcomes of mRCC patients from a tertiary academic medical center treated with empirically selected standard-of-care therapy. We utilized the Stanford Renal Cell Carcinoma Database (RCCD) to report on various outcome measures, including overall survival (OS) and the median number of lines of targeted therapies received from the time of metastatic diagnosis. We found that most metastatic patients did not survive long enough to attempt even half of the available targeted therapies. We also noted that patients who failed to receive a clinical benefit within the first two lines of therapy could still go on to experience clinical benefit in later lines of therapy. The term, “clinical benefit” was assigned to a line of therapy if a patient remained on drug treatment for three months or longer. Moreover, patients with clinical benefit in at least one line of therapy experienced significantly longer OS compared to those who did not have clinical benefit in at least one line of therapy. Developing biomarkers that identify patients who will receive clinical benefit in individual lines of therapy is one potential strategy for achieving rational drug sequencing in mRCC.

## 1. Introduction

Renal cell carcinoma (RCC) is the eighth most commonly diagnosed cancer in the United States, with 73,820 new cases and 14,770 related deaths estimated for 2019 [1,2,3]. Approximately 16% of patients present with de novo metastatic and often incurable disease, with a dismal five-year overall survival (OS) rate of 16% [2,4]. The therapeutic landscape of metastatic RCC treatment has dramatically evolved over the last few decades, with 15 different therapies now approved for the systemic treatment of metastatic disease. These drugs span a wide range of mechanisms of action, from cytokine therapy (interferon-alpha, interleukin-2); targeted therapy with small-molecule tyrosine kinase inhibitors (TKIs) of the vascular endothelial growth factor receptor (VEGFR) (axitinib, cabozantinib, lenvatinib, pazopanib, sorafenib, sunitinib) and kinase inhibitors of the mammalian target of rapamycin (mTOR) pathway (everolimus, temsirolimus); monoclonal antibodies directed against VEGF ligand (bevacizumab); and, more recently, immune checkpoint inhibitors targeting cytotoxic T-lymphocyte-associated protein 4 (ipilimumab), programmed death- ligand 1 (avelumab), and programmed cell death-1 receptor (nivolumab, pembrolizumab) [5,6,7,8,9,10,11].

This ever-expanding list of drugs from an already extensive range of options for metastatic renal cell carcinoma (mRCC) provides an opportunity to maximize the number of effective therapies that each patient receives, Recent Food and Drug Administration (FDA) approval of therapeutic regimens combining immune checkpoint inhibitors and TKIs only further emphasizes the need for biomarkers capable of identifying drugs that confer the longest duration of effect in individual patients. Moreover, as immunotherapy agents demonstrate overall survival (OS) benefits and clinical trials suggest that upfront cytoreductive nephrectomy is no longer superior to systemic therapy in patients with intermediate- or poor-risk prognoses, we anticipate an increasing shift in clinical practice toward a greater use of upfront systemic therapy [2,6,10,11,12,13,14,15].

Here, we investigated how the duration of each empirically selected therapy impacts outcomes. We report single-institution outcomes of drug therapy in mRCC using the Stanford Renal Cell Carcinoma Database (Stanford RCCD), a tertiary academic medical center database. The RCCD consists of clinico-demographic and outcomes data on localized and metastatic kidney cancer patients seen at Stanford from 2003 to the present [16,17]. We evaluated whether or not a patient experienced a clinical benefit for each therapy received and report on the mortality trends of these patients. “Clinical benefit” was assigned to a line of therapy if a patient remained on drug treatment for three months or longer (as assessed during a routine outpatient visit). Conversely, “lack of clinical benefit” was assigned to a line of therapy if a patient stopped drug treatment before three months (either because of tumor growth or adverse drug side effects). We chose three months because this timepoint has been used in previous RCC studies, including the CARMENA trial [13], to determine the clinical benefit of a given therapy. Furthermore, we wanted to investigate if the first clinical restaging, which is typically done three months after initiating a new therapy, correlated with overall survival.

We note that the vast majority of drugs received by the patients in our cohort were TKIs, and few received immuno-oncology therapy within our prespecified observation period. Upon analysis of this cohort, we present the following findings: First, we did not observe universal cross-resistance among the various drugs. Second, patients who received a greater number of overall therapies showed a trend toward longer OS, as did those patients who received a greater number of clinically beneficial therapies. Finally, patients who were maintained on a drug for a period of three months or longer (in at least one line of therapy) showed significantly longer OS compared to those who were not. Currently, without any biomarkers, approximately 50% of patients in the International Metastatic Renal Cell Carcinoma Database Consortium (IMDC) database never have a chance to receive second-line therapy [18]. Our data encourages the development of biomarkers with the goal of increasing clinical benefit received in all lines of therapy. Biomarkers that can predict benefit in early lines of therapy may enable patients to live longer, which in turn may allow them to receive and benefit from later lines of therapy.

## 2. Results

Among patients registered in the Stanford RCCD, we analyzed 194 mRCC patients with known death dates. According to the International Metastatic Renal Cell Carcinoma Database Consortium risk classification, 21 of these patients (11%) had a favorable-risk prognosis, and 173 (89%) had an intermediate- or poor-risk prognosis [19]. These 194 patients received a total of 504 independent lines of therapy, out of which 293 were beneficial (58%) per our definition of clinical benefit (see “Materials and Methods”) (see Table 1). As in other clinical settings, therapies given to patients were empirically selected based on drug availability and patient comorbidities. In general, sunitinib was most commonly given as a first-line therapy in 46.9% of patients, followed by pazopanib in 25.8% of patients and sorafenib in 15.5% of patients. Beyond that, there was significant heterogeneity in drug selection. See Table S1 and Figure S1 for specific drug frequencies and patterns.

The median number of therapies received per patient was two, while the median age at diagnosis was 60. The median OS of all-comers was 16.4 months, and the median therapy duration per patient was 9.92 months. Here, 139 patients were male (72%), and 55 patients were female (28%) (see Table 2).

In our study, 131 patients had clear-cell RCC, and 21 patients had non-clear-cell RCC, while the remaining 42 patients did not have an identified subtype. We compared patients with clear-cell and non-clear-cell RCC on the following parameters: Median overall survival, median number of therapies received, and median percentage of “clinically beneficial” therapies per patient (Table S2). As expected, patients with clear-cell RCC had a significantly higher median OS (*p* < 0.0001) and median percentage of “clinically beneficial” lines of therapy per patient (*p* = 0.008). There was no significant difference in the median number of lines of therapy received between the two groups (*p* = 0.4711).

**Figure 1:**
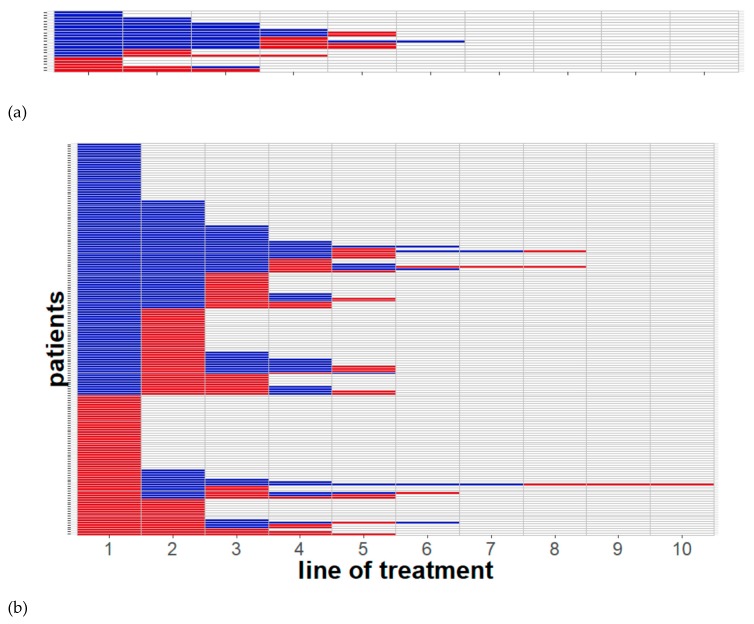
Heatmaps clustered by sequential therapy benefit showing a lack of cross-resistance among targeted therapies in mRCC (stratified by the International Metastatic Renal Cell Carcinoma Database Consortium (IMDC) prognostic risk score). Individual patients could still derive a clinical benefit despite failure in a previous line of therapy. (**a**) Favorable-risk prognosis treatment courses (21 patients); (**b**) intermediate- and poor-risk prognosis treatment courses (173 patients). Each row illustrates a single patient’s treatment course through a sequence of red and blue boxes from left to right. Each box shows one line of treatment (box size independent of therapy duration). Colors indicate therapy benefit (blue), lack of clinical benefit (red), or no further lines of treatment (gray) (for definitions, see “Materials and Methods”).

**Figure 2:**
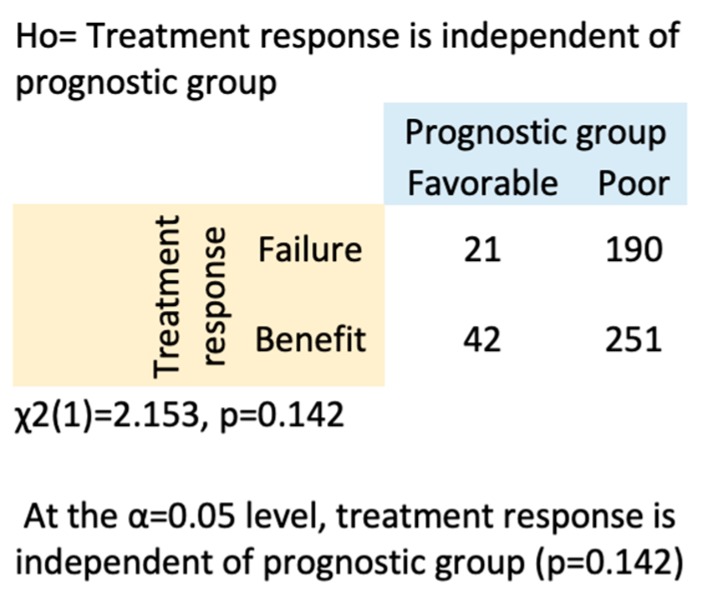
The clinical benefit in any given line was independent of the IMDC prognostic risk group. A chi-square analysis comparing treatment response in favorable- and intermediate- or poor-risk prognosis groups. In this analysis, intermediate and poor risk patients were combined; the combined group is labeled as, “Poor”.

Including all risk category patients, 53 patients (27.3%) experienced a benefit in every line of therapy received, while 49 patients (25.3%) experienced no clinical benefit in any line of therapy received (Figure 1). A total of 127 patients (65%) experienced a clinical benefit with first-line treatment. Heatmap analysis indicated that 42 patients (24%) with an intermediate- or poor-risk prognosis did not benefit from first-line treatment. A chi-square analysis of the full cohort demonstrated that clinical benefit did not depend on prognostic risk category (Figure 2). The median number of therapies received per patient was three in the favorable prognostic risk group and two in the intermediate/poor risk group. However, an unpaired *t*-test showed that this difference was not statistically significant (*p* = 0.238).

To address concerns regarding drug resistance in the context of agents having similar mechanisms of action, we examined how often patients received a clinical benefit from a later drug after failing a previous one. Despite drug failure in a prior line, 41 patients out of a total of 194 (21.1%) had a clinical benefit in a later line of targeted therapy (see Figure 1). Of note, five patients (2.6%) experienced their first clinical benefit in as late as the third line following two previous line failures.

**Figure 3:**
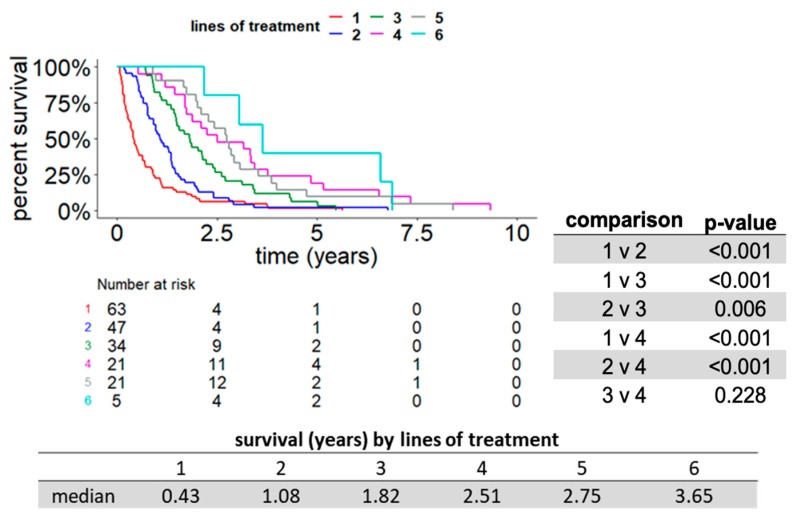
A greater number of total therapies received correlated with longer OS. Kaplan–Meier curves of OS are graphed. Patient cohorts in this figure are defined by the total number of treatment lines received (ranging from one to six). As only three patients received more than six lines of therapy, these patients were excluded. Subpanel: Pairwise statistical comparison of the length of OS between the four subgroups receiving one, two, three, and four lines of therapy.

**Figure 4:**
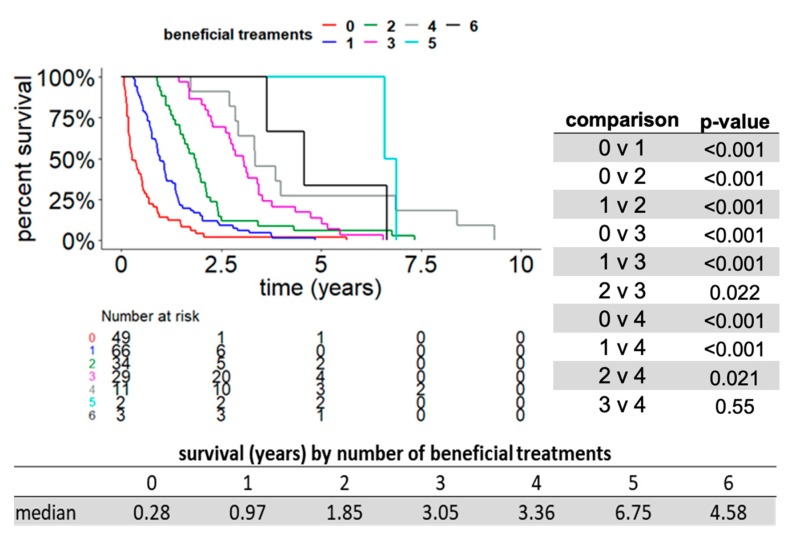
A greater number of beneficial therapies received correlated with longer OS. Kaplan–Meier OS curves for patient cohorts defined by the total number of beneficial lines of treatment received (independent of total number or duration of treatments). Subpanel: Pairwise statistical comparison of the duration of OS between the five subgroups receiving zero, one, two, three, and four beneficial lines of therapy.

Since most patients with metastatic RCC in our cohort were not able to receive more than two available therapies, we hypothesized that patients who were able to receive a greater number of therapies might have a longer overall survival. We examined whether total number of treatments received was correlated with overall survival using Kaplan–Meier analysis. We compared cohorts of patients that received a total of one, two, three, four, five, or six total therapies and compared the overall survival between cohorts in a pairwise fashion. This univariate analysis demonstrated that the total number of therapies received had a positive correlation with OS (Figure 3). For example, patients who received two lines of therapy had a statistically higher median overall survival (1.08 years) compared to those who received one line of therapy (0.43 years), *p* < 0.001. Next, we sought to determine whether the total number of beneficial therapies was correlated with overall survival using Kaplan–Meier analysis. We compared cohorts of patients that received zero, one, two, three, four, or five beneficial therapies and compared the overall survival between cohorts in a pairwise fashion. Our analysis showed that the number of beneficial therapies also showed a positive correlation with OS (Figure 4). For example, patients who received two lines of beneficial therapy had a statistically higher median overall survival (1.85 years) compared to those who received one line of beneficial therapy (0.97 years), *p* < 0.001.

**Figure 5:**
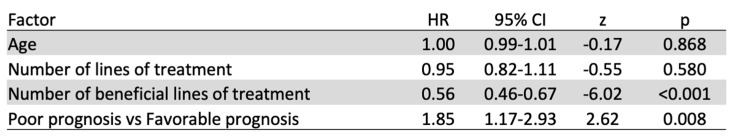
The number of beneficial lines of treatment and the IMDC risk prognostic score were both significantly correlated with OS. Cox regression multivariate testing of the relationship between the following factors: Age, number of treatment lines, number of beneficial lines of treatment, and intermediate- or poor-risk versus favorable-risk prognosis.

We next performed a multivariate analysis of OS using variables of age, total number of treatments, total number of beneficial treatments, and prognostic risk group. As expected, patients with more aggressive disease, characterized by intermediate or poor prognosis, had poorer survival outcomes compared to patients with favorable prognosis independent of age, total number of treatments, and total number of beneficial treatments (Figure 5). However, neither age nor total number of treatments was independently associated with OS. Interestingly, the total number of beneficial treatments showed a positive correlation with OS.

**Figure 6:**
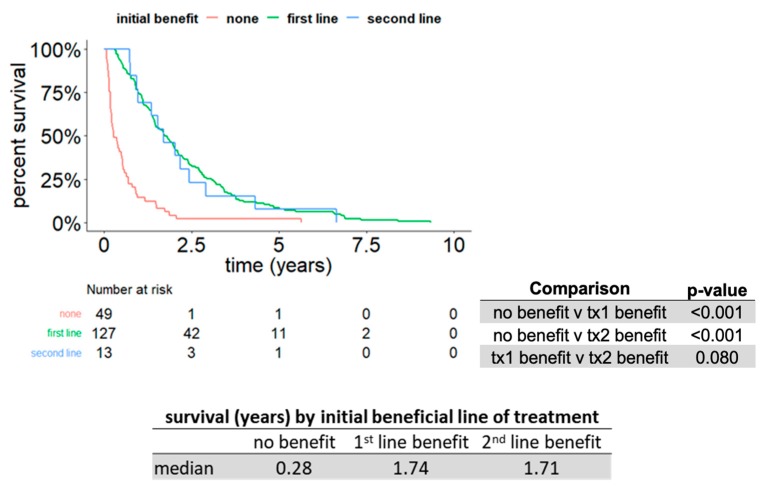
Patients maintained on a drug for at least three months in any line of therapy (within the first two lines) showed longer OS. Kaplan–Meier OS curves for all patients who received a minimum of two lines of targeted therapy stratified by timing of first clinical benefit (none, first-, or second-line). Subpanel: Statistical analysis showing no difference in the length of OS between first- and second-line therapies, but worse OS if patients could not be maintained on at least three months of drug treatment.

Finally, we explored whether the timing of a therapy benefit would impact OS (i.e., if a clinical benefit in the first or second lines would result in longer OS). We found that a clinical benefit occurring within either the first or second lines of therapy positively impacted OS compared to no benefit at all (*p* < 0.001). As long as patients remained on drug therapy for a period of at least three months in the first and/or second line (*p* = 0.08, Figure 6), median OS was greater. Larger cohorts of patients are needed to confirm the lack of significance in OS between first- and second-line benefit. However, our analyses highlight the importance of careful selection of agents that provide at least three months of clinical benefit as first or second-line treatment in mRCC patients.

In order to determine if our three-month “clinical benefit” correlated with an assessment of the “best clinical response” to each line of therapy, we compared these variables as follows. In our database, patients were classified as having either complete response (CR), partial response (PR), stable disease (SD), or progression of disease (PD) as the “best clinical response” during each line of therapy. The criteria used to assess “best clinical response” were in many cases based on formal Response Evaluation Criteria in Solid Tumors (RECIST tumor measurements), but in other cases they were based on a treating physician’s interpretation of imaging and clinical assessment without formal RECIST measurements. We had “best clinical response” measurements for 400 out of 504 lines of therapy. We divided these 400 clinical response measurements into two groups: Those with either CR, PR, or SD (called “response”) and those with PD (called “no response”). We performed a chi-square analysis to determine the association between our “clinical benefit” (yes vs. no) and clinical response measurements (response vs. no response) (Figure S2). Our results indicated that our definition of “clinical benefit” was, in fact, highly correlated with the clinical response criteria commonly used in practice settings (*p* < 0.0001).

## 3. Discussion

In this study, we report findings from a single-institution database on therapy outcomes in metastatic kidney cancer patients. This detailed look at real-world outcomes is particularly relevant in an era of a continuously expanding repertoire of targeted therapies and cytoreductive nephrectomy (CN) for patients with oligometastatic disease. While CN had shown an OS benefit in newly diagnosed mRCC patients in small randomized clinical trials compared to systemic interferon, this was not compared head-to-head against TKIs until the CARMENA trial, which narrowed the scope of upfront CN to metastatic patients with low overall disease burden and good performance status, thus elevating systemic therapy to the forefront of metastatic RCC management [13,19,20,21]. Currently, the treatment paradigm in mRCC is evolving from single-agent targeted therapy to combination regimens designed to maximize antitumor activity [8,9,15,22,23]. Combination therapy ultimately holds promise for potentially increasing long-term survival, but a major challenge now will be selecting appropriate combinations in the right sequence given the array of drugs currently available for use.

Prior studies looking at treatment patterns and clinical outcomes in mRCC patients have shown that patients who received second-line therapy demonstrated longer OS [3]. Furthermore, more granular studies of anti-vascular endothelial growth factor receptor (VEGFR) tyrosine kinase inhibitor (TKI) sequencing have indicated that there was no cross-resistance to a first-line anti-VEGFR TKI and that combined progression free survival (PFS) was generally longer for the sequence of sorafenib followed by sunitinib compared to sunitinib followed by sorafenib, supporting the rationale for treatment with a weaker TKI followed by a more potent TKI [24,25,26,27,28,29,30,31,32,33,34,35]. However, subsequent data provided by the SWITCH-I trial indicated that the combined PFS of two sequences (sunitinib followed by sorafenib or vice versa) showed no difference between treatment arms [34]. These trials ultimately raise additional questions about whether PFS is a valuable endpoint. Our study proposes setting a lower limit of three months’ response to any given treatment as an alternative method of correlating drug response to OS [36].

Our clinical cohort demographics closely matched those already reported in the literature [37]. Our cohort included patients whose median age, median OS, and median therapy duration in any line of treatment were similar to those demographics seen in clinical trials [38,39,40,41]. Our conclusions may thus be generalizable to a broader population. Our data corroborated prior studies showing no evidence for universal cross-resistance among targeted therapies, particularly VEGFR-directed TKIs. Moreover, we found significantly increased OS in patients who received more total lines of therapy and also a correlation of increased OS in patients with more beneficial lines of therapy. An expanded study of treatment sequencing in a larger cohort will be required to test whether sequential lines of beneficial therapy will ultimately lead to longer OS.

When investigating the timing of treatment benefit versus OS, we observed no significant difference between first- or second-line benefit, but being on drug therapy for at least three months did correlate with longer OS. It will be interesting to explore whether this phenomenon holds true in a larger cohort, particularly when looking at even later lines of therapy. In our study population, the percentage of patients deriving a benefit after failure was higher among intermediate- and poor-risk prognosis patients compared to good-risk prognosis patients (22.5% vs. 9.5% of patients receiving more than one line of therapy), which suggests that more aggressive disease alone may not increase the likelihood of intrinsic resistance to later lines of therapy. Our results might suggest that a three-month clinical benefit could be explored as a surrogate endpoint for either PFS or, more importantly, OS. If our results are upheld in larger studies, one approach to improve OS might suggest aggressive side effect management with a goal of remaining on each therapy for at least three months (as long as there is no sign of clinical progression) before switching agents.

Our results raise several hypotheses regarding the development of biomarkers for rational drug selection and sequencing. First, patients who only ever receive one line of ineffective therapy without an opportunity to try a second agent (18.5% of our cohort) are in greatest need of biomarkers. These patients develop rapid clinical progression and/or significant toxicity before completing three months of first-line treatment. If there were a way to allow this cohort to select a clinically beneficial therapy in the first line, this might impact overall survival. Alternatively, if there were an early molecular assessment biomarker to very quickly assess if tumors were progressing within days of initiating treatment, then greater efforts could be made to rapidly switch patients to a potentially effective second-line treatment, sparing them additional toxicity. A molecular or imaging biomarker of early therapeutic efficacy (i.e., before three-month imaging results) might be helpful in the general population to maximize the time spent receiving beneficial therapy and minimize the time spent without a clinical benefit [42,43]. It remains to be seen whether better biomarkers of therapeutic response can impact OS and whether aggressive tumor biology can be overcome with carefully selected therapy.

There were several limitations to our study. First, reporting OS statistics in our cohort necessitated the exclusion of patients who remained alive after the designated cut-off date. The excluded patients had access to a greater number of therapies, as agents continued to be FDA-approved in later years (see Table S1 and Figure S2). Specifically, few patients received drugs such as lenvatinib, cabozantinib, or ipilimumab. However, we believe our results are relevant, because while TKIs such as cabozantinib have shown an OS benefit, they are not curative. Another limitation was the possibility that lines classified as “lack of clinical benefit” included patients who stopped therapy to undergo surgery for oligometastatic disease or due to intolerable side effects. It is possible that some of these individuals could have received longer duration of drug therapy with aggressive side effect management. We were not able to examine dose intensity, and as dose may also impact clinical benefit, it will be important to note this factor in future studies. Therefore, in future investigations, it will be important to look at an expanded cohort (such as multi-institutional data) to correlate clinical benefit versus lack of benefit with standard tumor response imaging and identify those who stop therapy because of adverse effects. Finally, the slightly higher percentage of patients who had progressive disease after first-line therapy was higher than that reported in clinical trial populations. We suspect this may have been the result of the smaller number of individuals considered, and if the study were further expanded to all patients currently alive, this percentage would be more consistent with those reported in the literature.

Our observations are especially pertinent in the era of immunotherapy, since the failure of such agents means that practitioners will continue to rely on VEGFR-targeted TKIs or mTOR inhibitors to control disease post-immunotherapy. Evidence of little-to-no universal cross-resistance among TKIs suggests that oncologists need an objective means for guiding an individual patient through multiple potentially effective therapies before irreversible clinical decline. A major consideration for applying this strategy will be to test whether such biomarkers lead to the ultimate prolongation of OS. Our conclusions ultimately point to an urgent need for prioritizing biomarker research in mRCC in order to improve overall survival, particularly for patients with primary drug refractory disease.

## 4. Materials and Methods

### 4.1. Patients

A retrospective, noninterventional study was conducted using data from the Stanford RCCD. To be eligible, patients were required to meet each of the following inclusion criteria:

(i) Have histologically confirmed locally advanced or metastatic RCC;

(ii) Have started systemic therapy with one of the following agents between 1 January 2003 and 16 July 2017: Axitinib, bevacizumab, cabozantinib, everolimus, interferon-alpha, interleukin-2, ipilimumab, lenvatinib, nivolumab, pazopanib, sorafenib, sunitinib, or temsirolimus;

(iii) Age ≥ 18 at the time of RCC diagnosis;

(iv) Have a confirmed death date.

Patients treated as part of clinical research trials were included when calculating the median number of lines of therapy received and survival analyses.

### 4.2. Data Source

The Stanford RCCD is a database that captures patients diagnosed with localized and metastatic kidney cancer who were seen at the Stanford Cancer Institute. The RCCD was approved by the institutional review board at Stanford School of Medicine, protocol 7981: “Outcomes for Kidney Tumors”, IRB registration #4593. This study was conducted in accordance with the principles of the Declaration of Helsinki and the International Conference on Harmonisation guidelines for Good Clinical Practice.

### 4.3. Outcome Variables

Sex and age were recorded as demographic measures. Patients were stratified into prognostic risk groups according to their International Metastatic Renal Cell Carcinoma Database Consortium (IMDC) score [5,18,44,45,46,47,48]. Patients with an IMDC score of zero were designated as favorable risk, while patients with scores equal or greater than one was designated as intermediate/poor risk. A label of clinical benefit was assigned to a line of therapy if a patient remained on drug treatment for three months or longer (as assessed during a routine outpatient visit). Conversely, lack of benefit was assigned to a line of therapy if a patient stopped drug treatment before three months’ time (either because of tumor growth or adverse drug side effects). OS was determined from the start of first systemic therapy, not the original cancer diagnosis date.

### 4.4. Statistical Analysis

Cox regression was used to assess the effect of categorical and continuous variables on survival. The effect of each factor on OS was further evaluated by the Kruskal–Wallis test for nonparametric data and Dunn’s test for post hoc comparisons. Here, *p*-values were adjusted using the Benjamini–Hochberg correction for multiple comparisons [49]. Kaplan–Meier plots depict survival probability. The “number at risk” indicates patients who were still alive at the end of each timepoint. The survival curves generated for the different patient groups were statistically compared in a pairwise fashion with a log-rank test (a type of chi-square test) in R. Statistical significance was set at the level of α < 0.05. Results are presented as median and interquartile range or as count (in percent). Statistical analyses were performed in R [50] or GraphPad Prism (San Diego, CA, USA).

## 5. Conclusions

Our study is a retrospective review reporting findings from a single-institution database on therapy outcomes in metastatic kidney cancer patients receiving targeted therapy. We found that there was no direct evidence of universal cross-resistance among multiple targeted therapy options in mRCC. Moreover, we discovered a trend of longer OS in patients who received more total lines of therapy, which continued to hold true when limiting observations to only beneficial lines. Additionally, we discovered that maintaining patients on drug treatment for at least three months in any line of therapy was more critical than the specific timing of such a benefit. This finding is particularly relevant for patients who only receive one line of therapy before succumbing to their disease. Larger studies will be needed to confirm these observations, but our findings suggest that efforts to develop biomarkers reporting on early therapeutic efficacy could maximize the length of time patients spend receiving effective treatment in each line.

## Figures and Tables

**Table 1:** Summary of demographics, treatment line, and overall survival (OS) data of the cohort of 194 metastatic renal cell carcinoma (mRCC) patients.

Parameter	Median	Range
Age (years)	60	(18–91)
Therapy lines/patient	2.0	(1–10)
Beneficial therapy lines/patient	1.0	(0.0–6.0)
Individual therapy line duration (months)	9.9	(0.03–70.1)
OS (months)	16.4	(0.83–113.5)

**Table 2:** Summary of gender distribution for the cohort of 194 mRCC patients.

Sex	*n*	%
Male	139	72
Female	55	28

## Supplementary Materials

The following are available online at https://www.mdpi.com/2072-6694/11/7/1000/s1, Table S1: Drug utilization in the cohort of 194 mRCC patients, Figure S1: Heatmap demonstrating the specific sequence of drugs used for each patient in the cohort of 194 mRCC patients, Figure S2: Three-month clinical benefit correlates with best clinical response during treatment, chi-squared analysis, Table S2: Median Overall Survival (OS) significantly higher for patients with clear cell RCC vs non clear cell RCC.
