# Supplementary material for: Time on Therapy for at Least Three Months Correlates with Overall Survival in Metastatic Renal Cell Carcinoma
Source: Cancers (Basel). 2019 Jul 17;11(7):1000. doi: 10.3390/cancers11071000 (PMC6678132; doi:10.3390/cancers11071000)
Supplement: Supplementary file 1 [file cancers-11-01000-s001.pdf]

# Supplementary Materials: Time on Therapy for at Least Three Months Correlates with Overall Survival in Metastatic Renal Cell Carcinoma

**Viola J. Chen, Gabriela Hernandez-Meza, Prashasti Agrawal, Chiyuan A. Zhang, Lijia Xie, Cynthia L. Gong, Christian R. Hoerner, Sandy Srinivas, Eric K. Oermann and Alice C. Fan**

## Supplementary Tables and Figures

A detailed overview on drug utilization frequency in the cohort of metastatic RCC patients. The histogram and Table S1 show how many times among the 504 independent lines of therapy each drug was received.

**Table S1.** Drug utilization in the cohort of 194 mRCC patients. "Other" indicates an investigational agent received on a clinical trial.

| Name of Drug | Frequency |
|---|---|
| Sunitinib | 116 |
| Pazopanib | 102 |
| Everolimus | 69 |
| Sorafenib | 59 |
| Axitinib | 42 |
| Temsirolimus | 36 |
| Bevacizumab | 28 |
| Nivolumab | 25 |
| Other | 18 |
| Cabozantinib | 4 |
| Interleukin-2 | 3 |
| Ipilimumab | 1 |
| Lenvatinib | 1 |
| Total | 504 |

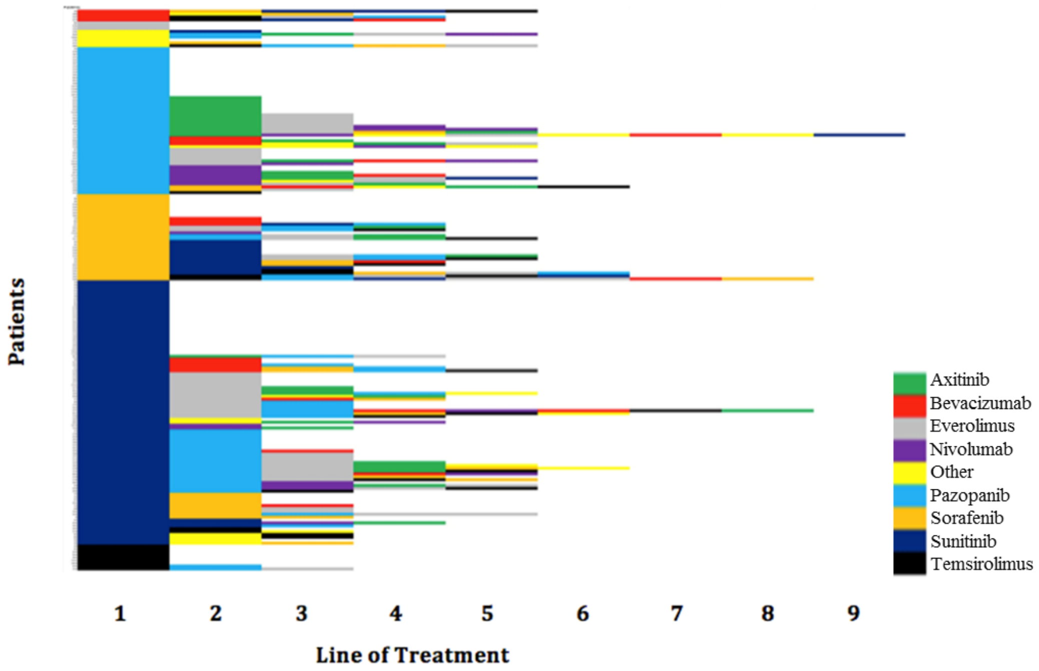


**Figure S1.** Heatmap demonstrating the specific sequence of drugs used for each patient in in the cohort of 194 mRCC patients. The 9 most commonly used drugs are shown individually, and the rest are included in the category of "Other", which includes investigational agents, cabozantinib, interleukin-2, ipilimumab, lenvatinib.

| | | Best Clinical Response | |
|---|---|---|---|
| | | Response | No Response |
| Clinical Benefit | Yes | 215 | 61 |
| | No | 29 | 95 |

$\chi 2 = 106.9$, $p < 0.0001$

**Figure S2.** Three-month clinical benefit correlates with best clinical response during treatment, Chi squared analysis. Best clinical response assessments were recorded for 400 lines of therapy and were divided into two groups: those with complete response, partial response, or stable disease (called response) and those with progressive disease (called no response). Chi square analysis to determine the association between our "clinical benefit" (yes vs. no) and best clinical response measurements (response vs no response).

**Table S2.** Median Overall Survival (OS) significantly higher for patients with clear cell RCC vs non clear cell RCC. Unpaired T-tests comparing clear cell vs non-clear cell RCC patients show significant difference in median percentage of "clinically beneficial" therapies but no significant difference in median number of therapies received.

| Type of RCC | Median OS (Months) | Median Number of Lines of Therapy | Median Percentage of "Clinically Beneficial" Lines of Therapy Per Patient |
|---|---|---|---|
| **Clear Cell (*n* = 131)** | 20.4 | 2 | 0.60 |
| **Non-Clear Cell (*n* = 21)** | 11.4 | 2 | 0.33 |
| **Comparison** | $p < 0.0001$<br>Log-Rank Test | $p = 0.4711$<br>Unpaired *t*-test | $p = 0.008$<br>Unpaired *t*-test |

© 2019 by the authors. Licensee MDPI, Basel, Switzerland. This article is an open access article distributed under the terms and conditions of the Creative Commons Attribution (CC BY) license (http://creativecommons.org/licenses/by/4.0/).
